# Incidence and influencing factors of acute gastrointestinal injury after cardiac surgery

**DOI:** 10.1186/s12872-023-03475-6

**Published:** 2023-09-01

**Authors:** Ruolin Lu, Biwen Yang

**Affiliations:** 1 Department of Clinical Nutrition, the First Affiliated Hospital of Soochow University, Suzhou, Jiangsu Province China; 2 Department of Cardiovascular Surgery, the First Affiliated Hospital of Soochow University, No. 899, Pinghai Road, Sujin Street, Suzhou, 215000 Jiangsu China

**Keywords:** Cardiac surgical procedures, Acute gastrointestinal injury, Nutrition, Influencing factors

## Abstract

**Background:**

To investigate the incidence and influencing factors of acute gastrointestinal injury (AGI) after cardiac surgery.

**Methods:**

A total of 346 cases receiving treatment in the Intensive Care Unit (ICU) of the Department of Cardiovascular Surgery in our hospital from January 2021 to December 2021 were enrolled and their basic information was collected, including age, gender, height, weight, past medical history, Nutrition Risk Screening 2002, Body Mass Index (BMI), total operation duration, stay in ICU, preoperative blood routine examination results, complete biochemical examination, diamine oxidase (DAO) on Day 1, D-lactic acid index, a postoperative gastrointestinal condition, other postoperative complications and death during hospitalization. Moreover, logistic regression analysis was performed to identify the independent risk factors influencing the incidence of AGI after cardiac surgery.

**Results:**

The incidence and mortality of AGI after cardiac surgery were 10.40% (36/346) and 25% (9/36), respectively. A dichotomous logistic regression multivariate analysis revealed that DAO on Day 1 (odd ratio = 1.062, *p* = 0.006) and stay in ICU (odd ratio = 1.192, *p* < 0.001) were independent risk factors of AGI after cardiac surgery, and total protein is a protective factor (odd ratio = 0.914, *p* = 0.012).

**Conclusions:**

Factors influencing AGI after cardiac surgery have been determined in this study. Our data suggest that patients with AGI after cardiac surgery have a decreased preoperative total protein, and elevated DAO on Day 1. Total protein and DAO on Day 1 were found to be correlated with AGI.

## Introduction

Gastrointestinal (GI) complications after cardiac surgery, although not the most common, need to be detected early because they are associated with high mortality rates. It has been demonstrated that the incidence and mortality of gastrointestinal complications after cardiac surgery are in the range of 1.21-4.17% [1–4] and 13.9-52%, respectively [5, 6]. Acute GI injury (AGI) is the malfunctioning of the GI tract in critically ill patients due to their acute illness [7]. The relationship between AGI or other gastrointestinal dysfunctions and the occurrence of gastrointestinal complications following cardiac surgery remains inadequately understood. Patients undergoing cardiac surgery are vulnerable to gastrointestinal dysfunction, primarily due to the diminished blood supply to the gastrointestinal tract resulting from surgical trauma.These disruptions can profoundly impact patient prognosis, underscoring the need for further research on this domain [8]. Therefore, the recovery of gastrointestinal function after cardiac surgery is essential to improve patients’ prognosis, and medical staff should take AGI seriously. In this study, a retrospective analysis of 346 cardiac surgery cases was performed to investigate the incidence of AGI and explore its influencing factors. Early intervention of the factors influencing AGI may improve the prognosis of patients who have undergone cardiac surgery.

## Materials and methods

### Demographic characteristics of the enrolled patients

A total of 346 patients hospitalized in the Cardiac and Vascular Surgery Monitoring Unit of the First Affiliated Hospital of Soochow University from January 2021 to December 2021 were retrospectively selected and analyzed. These patients included 206 males and 140 females, with an average age of 56.07 ± 13.35 years old, The minimum age was 18 and the maximum age was 79. Inclusion criteria: At least 18 years old; no history of blood transfusion, albumin, or special nutritional support before admission; and complete clinical data. Exclusion criteria: age < 18 years old; history of blood transfusion, albumin, or special nutritional support before admission; and a history of gastrointestinal tumor or resection. The study was approved by the hospital’s ethics committee and all patients voluntarily participated and signed the informed consent form.

### Monitoring and evaluation of gastrointestinal function

This study investigated cases of AGI, which manifested as nausea, vomiting, hypoactive bowel sounds, paralytic ileus, diarrhea, gastroparesis with gastric retention, gastrointestinal bleeding, abdominal distention, intestinal bleeding or perforation, and other symptoms. The AGI diagnostic criteria [7]: AGI level 1, with the risk of gastrointestinal dysfunction and failure; AGI level 2, gastrointestinal dysfunction; AGI level 3: gastrointestinal failure; AGI level 4, gastrointestinal failure with distant organ dysfunction. The diagnosis of AGI was based on the clinical characteristics and examination results.

### Observation indicators

The records of the patient’s preoperative medical history included their age, gender, height, weight, BMI, Nutrition Risk Screening 2002, preoperative blood routine examination results, complete biochemical examination results, operation type, total operation duration, diamine oxidase (DAO) on Day 1, D-lactic acid index, stay in ICU and death during hospitalization.

### Statistical method

Data were analyzed using SPSS 22.0 statistical analysis software. Measurement data were expressed as mean ± standard deviation ($\stackrel{-}{x}$ ± s). Data not conforming to normal distribution were analyzed using a *t-test*, and data not conforming to normal distribution were analyzed by a non-parametric rank sum test. Count data were presented as cases (the percentage), and inter-group comparisons were performed using the χ^2^ test. Logistic binary regression analysis was performed to explore the factors influencing the incidence of AGI after cardiac surgery. *p* < 0.05 was considered statistically significant.

## Results

### General information

Among the 346 patients who underwent cardiac surgery, 206 were male and 140 were female. The average age was 56.07 ± 13.35 years old. Demographic data of the patients are presented in Table 1. Furthermore, 36 patients developed AGI during the hospitalization, with an incidence of 10.40% (36/346) and a mortality rate of 25% (9/36). This was higher than the 1.9% (6/310) mortality of non-AGI patients (p < 0.001, see Tables 1 and 2). There were 13 cases of septic shock and 171 cases of pulmonary infection after the operation, and 18 cases received CRRT treatment after the operation.

**Table 1:** The basic clinical data of the 346 enrolled cases

Classification	n (%)
Gender (M)	206 (59.5)
Smoking	53 (15.3)
Drinking	30 (8.7)
NRS 2002 score	
< 3	325 (93.9)
3	19 (5.5)
4	1 (0.3)
5	1 (0.3)
Hypertension	148 (42.8)
Diabetes	31 (9.0)
Apoplexy	17 (4.9)
Chronic kidney disease	7 (2.0)
Operation type	
TA-TAVI	3 (0.9)
Valve replacement	175 (50.6)
Open aortic surgery	64 (18.5)
Modified morrow	2 (0.6)
CABG surgery	39 (11.3)
Congenital heart disease surgery	42 (12.1)
Cardiac exfoliation	7 (2.0)
Cardiac tumor surgery	14 (4.0)
Postoperative AGI	36 (10.4)
Death	15 (4.3)

Abbreviations: NRS = NRS Nutrition Risk Screening, TA-TAVI = Transapical Transcatheter Aortic Valve Implantation, CABG = Coronary Artery Bypass Grafting, AGI = Acute Gastrointestinal Injury

**Table 2:** Mortality of the subjects involved (n, %)

	Cases (n)	Death cases	Survival cases	X2	P value
AGI	36	9 (25.0)	27 (75.0)	41.372	< 0.001
Non-AGI	310	6 (1.9)	304 (98.1)		
The total	346	15 (4.3)	331 (95.7)		

Abbreviations: AGI = Acute Gastrointestinal Injury

### Univariate analysis of 346 cardiac surgery cases

AGI was detected in 36 out of 346 cases of cardiac surgery (incidence = 10.40%). AGI and non-AGI were consideted to be dependent variables in the influencing factor analysis. The univariate analysis showed that there were statistically significant differences between the two groups in terms of chronic kidney disease history, nutritional indicators (total protein, prealbumin, creatinine, red blood cells, lymphocytes, hemoglobin), intestinal barrier function indicators (DAO, D-lactate), operation type, operation duration, and stay in the ICU (*p* < 0.05, see Table 3).

**Table 3:** Univariate analysis of the incidence of AGI after cardiac surgery in 346 enrolled cases

Factors	AGI (n = 36)	Non-AGI (n = 310)	t/χ ^2^	*p* value
Age (years, $\bar x \pm s$)	58.81 ± 13.50	55.75 ± 13.32	1.301	0.194
Gender, n (%)				
Male	26 (72.2)	180 (58. 1)		
Female	10 (27.8)	130 (41.9)	2.684	0.07
Smoking, n (%)	8 (22.2)	45 (14.5)	1.477	0.165
Drinking, n (%)	2 (5.6)	28 (9.0)	0.151	0.373
BMI (kg/m^2^)	24.19 ± 4.25	24.22 ± 3.73	0.048	0.962
Previous history, n (%)				
Hypertension	16 (44.4)	132 (42.6)	0.046	0.483
Diabetes	4 (11.1)	27 (8.7)	0.228	0.407
Apoplexy	0	17 (5.5)	2.706	0.144
Chronic kidney disease	3 (8.3)	4 (1.30)	8.072	0.027
LVEF (%)	59.78 ± 7.61	56.23 ± 9.85	1.048	0.297
CPB	32 (88.9)	264 (85.2)	0.124	0.725
Laboratory test				
Total protein (g/L)	60.98 ± 5.83	64.50 ± 5.92	3.38	0.001
Albumin (g/L)	37.69 ± 3.37	38.93 ± 3.75	1.892	0.059
Prealbumin (mg/L)	224.93 ± 61.96	244.90 ± 52.70	2.112	0.035
Creatinine (umol/L)	140.95 ± 155.17	79.14 ± 70.33	2.362	0.024
Red blood cell count (10^12/L)	4.17 ± 0.63	4.41 ± 0.58	2.283	0.023
Lymphocyte count (10^9/L)	1.18 ± 0.51	1.55 ± 0.65	3.314	0.001
Hemoglobin (g/L)	121.69 ± 18.63	129.25 ± 17.91	2.312	0.018
DAO (U/L)	16.09 ± 9.98	12.27 ± 8.92	2.403	0.017
D- lactate (mg/L)	28.84 ± 8.41	24.89 ± 8.77	2.567	0.011
Operation duration, min	437.06 ± 163.62	336.75 ± 103.14	3.596	0.001
Stay in ICU, d	16.17 ± 13.97	5.88 ± 3.39	10.628	< 0.001

Abbreviations: NRS = Nutrition Risk Screening, TA-TAVI = Transapical Transcatheter Aortic Valve Implantation, CABG = Coronary Artery Bypass Grafting, AGI = Acute Gastrointestinal Injury, LVEF = Left Ventricular Ejection Fraction, DAO = Diamine Oxidase, ICU = Intensive Care Unit

### Multivariate analysis of the incidence of AGI after cardiac surgery

A binominal logistic regression was performed on the factors with *p* < 0.05 in the univariate analysis as independent variables. For binary independent variables, the influencing factor was coded as 0 if it did not exist and 1 if it did. For the quantitative independent variables, we inputted the original values of the nutritional index, intestinal function index, total operation time, and stay in ICU. Additionally, dummy variables were set when the operation type was unordered multi-classification (Table 4). The dichotomous logistic stepwise regression analysis showed that total protein, DAO, and stay in ICU were significant independent factors for AGI (*p* < 0.05, Table 5)

**Table 4:** Assignment of independent variables in binominal logistic regression analysis

Independent variable	Assignment criteria
Chronic kidney disease	No = 0; Yes = 1
Operation type	valve replacement = 1, open aortic surgery = 2, other = 3

**Table 5:** Multivariate analysis of the incidence of AGI after cardiac surgery in 346 enrolled cases

Classification	OR	95% CI	*p*
Operation duration (min)	1.003	(1.000-1.006)	0.067
Total protein (g/L)	0.914	(0.852–0.980)	0.012
DAO (U/L)	1.062	(1.017–1.110)	0.006
Stay in ICU (d)	1.192	(1.104–1.287)	< 0.001

Abbreviations: DAO = Diamine Oxidase; ICU = Intensive Care Unit

## Discussion

### AGI after cardiac surgery is a rare complication with high mortality [3–5]

Gastrointestinal injuries are affected by various factors before and during cardiac surgery [9], with the major cause being splanchnic hypoperfusion [10]. In addition, systemic inflammation also contributes to the occurrence of GI complications [11]. Several nonischemic mechanisms may contribute to GI complications, including bacterial translocation, adverse drug reactions, and iatrogenic organ injury [8]. Preoperative hypertension, renal failure, chronic lung disease, and heart failure history have been identified as independent risk factors for acute mesenteric ischemia or postoperative gastrointestinal bleeding after cardiac surgery [9]. However, disease history was not found to be an independent risk factor in this study. Out of the 346 cases included in the study, the incidence of AGI after cardiac surgery was 10.4% (36/346), with a mortality of 25% (9/36). Dichotomous logistic regression showed that patients with higher preoperative total protein had lower risks of complications (OR = 0.914, 95%CI 0.852–0.980, *p* = 0.012). Total protein in the serum is a crucial indicator for monitoring nutritional status [12], and patients with extremely low levels of total protein have a low survival rate and increased risk of postoperative infection [13]. Preoperative nutritional status is a factor that significantly affects the prognosis of AGI. Malnutrition can cause systemic multiple-organ damage and renal insufficiency, which places an additional burden on the heart, leading to malignant circulation, prolonged stay in ICU, adversely affecting the prognosis, and even leading to death. Patients with a longer stay in ICU was associated with a higher risk of AGI after cardiac surgery (OR = 1.192, 95%CI 1.104–1.287, *p* < 0.001). One trial showed that providing various nutritional support measures can improve caloric deficiency and reduce mortality and complications in patients undergoing elective cardiac surgery [14].

### DAO was an independent risk factor of the incidence of AGI after cardiac surgery (OR = 1.062, 95%CI 1.017–1.110, ***p*** = 0.006)

DAO is a highly active intracellular enzyme found in the cytoplasm of villus cells in the upper intestinal mucosa. When the intestinal mucosa is damaged, DAO is transported via the lymphatic and blood vessels in the intestinal intercellular space [15, 16]. Ye et al. [17] found that the level of DAO in the plasma was positively correlated with the degree of gastrointestinal dysfunction in critically ill children. Several studies [16, 18] have demonstrated that the D-lactate concentration in the blood is positively correlated with the severity of intestinal mucosal barrier damage, and early DAO specificity increases in patients with intestinal mucosal injury. Thus, elevated DAO in the serum is a sensitive indicator of intestinal mucosal barrier integrity. The DAO test is convenient for clinicians, is minimally traumatic for patients, and has a low cost. Therefore, the level of DAO can serve as a good indicator of the incidence of AGI after cardiac surgery. In addition to the function of digestion and absorption, the gastrointestinal tract also has the most important intestinal barrier function. Normal intestinal mucosa acts as a barrier that prevents entry of toxins and other substances, thus alleviating intestinal absorption. However, if gastrointestinal barrier function is damaged, it increased, and DAO increases. Additionally, changes in the intestinal microenvironment can cause intestinal flora disorder, resulting in the translocation of many intestinal bacteria and toxins, further exacerbating systemic inflammatory reactions [19]. Gastrointestinal failure can significantly increase mortality in severe cases [19, 20]. The mechanism underlying gastrointestinal barrier dysfunction in AGI after cardiac surgery may include the following: (1) Mechanical barrier dysfunction: is caused by the traction and compression of the heart during extracorporeal and non-extracorporeal circulation, leading to decreased gastrointestinal blood perfusion and ischemia-reperfusion injury. AGI is one of the clinical manifestations of ischemia-reperfusion injury [21]. (2) Chemical barrier dysfunction: is due to long-term fasting and total parenteral nutrition resulting in a decrease in gastric acid, bile, lysozyme, mucopolysaccharide, and other chemical components. (3) Biological barrier dysfunction: is caused by the long-term and massive use of broad-spectrum antibiotics, which leads to a reduction in the colonization of normal flora, an increase in pathogenic bacteria, and an increase in the permeability of the intestinal mucosa,compared with healthy individuals. Furthermore, the presence of a large amount of flora can disturb the internal environment and inhibit the immune system function [22]. (4) Immune barrier dysfunction: this is caused by shock, infection, trauma, and other factors, which damage the intestinal immune function and create potential niches for pathogenic bacteria. Furthermore, the integrity of regulatory cells can be damaged when the intestinal flora is disrupted [23].

## Conclusion

The incidence of AGI after cardiac surgery is low but associated with a high rate of mortality. Close surveillance, prompt diagnosis, and adequate interdisciplinary treatment of AGI are important approaches for reducing mortality rates. Our data suggest that decreased preoperative total protein and elevated DAO on Day 1 are associated with the incidence of AGI after cardiac surgery. Understanding this relationship and referencing these data during the perioperative period may help optimize perioperative prevention and treatment strategies.

## Data Availability

All data generated or analyzed during this study are included in this published article.
